# Bethlem Hospital

**Published:** 1852-10-01

**Authors:** 


### BETHLEM HOSPITAL.

We must consider Dr. Hood's nomination to the important and distinguished position of physician to Bethlem Hospital as *un fait accompli.* We do not, therefore, feel called upon to refer either in terms of animadversion or eulogy to the proceedings of the late contested election. By a large majority of votes this gentleman has been selected to preside over the medical department of this large national asylum. We sincerely trust, Dr. Hood may realize the kind anticipations of his friends, and perform with satisfaction to his own conscience as well as to the public, the serious and responsible duties of the important office to which he has been appointed. It will always afford the editor of this journal much pleasure to support him in his efforts to promote the interests of Bethlem Hospital, the welfare of the unhappy patients confined within its walls, and the advancement of medico-psychological science.

